# Immunosuppressive Drugs Modulate the Replication of Hepatitis B Virus (HBV) in a Hydrodynamic Injection Mouse Model

**DOI:** 10.1371/journal.pone.0085832

**Published:** 2014-01-21

**Authors:** Junzhong Wang, Baoju Wang, Shunmei Huang, Zhitao Song, Jun Wu, Ejuan Zhang, Zhenni Zhu, Bin Zhu, Ying Yin, Yong Lin, Yang Xu, Xin Zheng, Mengji Lu, Dongliang Yang

**Affiliations:** 1 Department of Infectious Diseases, Union Hospital, Tongji Medical College, Huazhong University of Science and Technology, Wuhan, China; 2 Institute of Virology, University Hospital Essen, University of Duisburg-Essen, Germany; 3 Department of Microbiology, Tongji Medical College, Huazhong University of Science and Technology, Wuhan, China; Drexel University College of Medicine, United States of America

## Abstract

Hepatitis B virus (HBV) reactivation and recurrence are common in patients under immunosuppression and can be controlled by hepatitis B immunoglobulin, antivirals, and hepatitis B vaccine. However, the detailed analysis of HBV infection under immunosuppression is essential for the prophylaxis and therapy for HBV reactivation and recurrence. In this study, HBV replication and T cell responses were analyzed in a HBV-transfected mouse model under immunosuppressive therapy. During the treatment, HBV replication was at a high level in mice treated with dexamethasone, cyclosporine, and cyclophosphamide, whereas was terminated in mice treated with mycophenolate mofetil. After the withdrawal, HBV replication was at low or high levels in the dexamethasone-treated mice or in both cyclosporine- and cyclophosphamide-treated mice. The early withdrawal of cyclosporine allowed the recovery of suppressed T cell responses and led to subsequent HBV clearance, while the adoptive immune transfer to the mice with HBV persistence led to HBV suppression. Taken together, long-term HBV persistence under immunosuppression depends on the immunosuppressive drugs used and on the treatment duration and is mediated by the suppressed intrahepatic CD8 T cell response. These data may be helpful for individualized immunosuppressive therapy in patients with high risk of HBV reactivation and recurrence, and the mouse system is suitable for studying HBV reactivation and recurrence under immunosuppression.

## Introduction

Hepatitis B virus (HBV) infection is widely distributed throughout the world. At least 350 million people are HBV carriers and are at high risk for developing hepatic decompensation, cirrhosis, and hepatocellular carcinoma [1]. Strong and polyclonal CD8+ and CD4+ T cell responses are essential for the clearance of HBV infections from the liver[2]; therefore, patients receiving immunosuppressive therapy may have a potential high risk for HBV infection due to the lack of adequate immunity. HBV reactivation under immunosuppressive therapy occurs frequently in patients with chronic and resolved HBV infection and is rarely reported in HBV seronegative patients [3]. HBV recurrence after liver transplantation occurs in 80–100% of patients without any prevention, while only occurring in up to 6.1% of patients after prophylactic treatment using hepatitis B immunoglobulin (HBIG) and nucleoside/nucleotide analogs (NAs) [4]. Although HBIG, NAs, and the hepatitis B vaccine are effective for preventing and treating HBV reactivation and recurrence, many factors remain unresolved. For example, patients under immunosuppressive therapy have poor response rates to the hepatitis B vaccine [5], [6], and the strategies for the prophylaxis and treatment of HBV reactivation and recurrence should be optimized. Detailed analysis of the HBV infection under immunosuppression is essential for resolving the above-mentioned problems.

For HBV research, useful mouse models with transient and persistent HBV replication were established based on a technique called hydrodynamic injection (HI) [7], [8] and was used to assess the effectiveness of vaccines and to examine the relationship between the types of immune responses and HBV clearance [9]–[11]. Given that HI of pAAV/HBV1.2 in BALB/c mice leads to transient HBV replication and gene expression in the liver [8], [9], this system could be used to analyze the influence of the immunosuppressive agents on HBV replication. Four widely used immunosuppressive drugs were selected: 1) corticoid: dexamethasone (DEX), 2) calcineurin inhibitor: cyclosporine A (CsA), 3) alkylating agents: cyclophosphamide (CYP), and 4) antimetabolite: mycophenolate mofetil (MMF). Using the immune competent mouse model after HBV HI, we demonstrated that HBV replication under immunosuppressive therapy was extended by the treatment with immunosuppressive agents and that transient treatment with CsA and CYP led to long-term HBV persistence with a high level of viral replication. Notably, the mRNA levels of the T cell markers and cytokine expression in the liver were significantly suppressed by CsA treatment and were restored by the early withdrawal of CsA, indicating that an impaired immunological function in the hepatic compartment leads to HBV persistence. Furthermore, adoptive immune transfer led to HBV suppression.

## Materials and Methods

### Animals

Female BALB/c mice at 6–8 weeks of age were purchased from the Centers for Disease Control, Hubei, China, and were kept under specific pathogen-free conditions in the Experimental Animal Centre of Tongji Medical College, Huazhong University of Science and Technology.

This study was performed in strict accordance with the recommendations in the Guide for the Care and Use of Laboratory Animals of the National Institutes of Health. The protocol was approved by the Institutional Animal Care and Use Committee at Tongji Medical College, Huazhong University of Science and Technology (Permit Number: IACUC-2010-268). All surgeries were performed under sodium pentobarbital anesthesia, and every effort was made to minimize suffering.

### Treatment with immunosuppressive agents

Immunosuppressive agents or saline were administered intraperitoneally 1 week prior to the HI at the following doses in a volume of 0.25 ml: DEX (Hubei Tianyao pharma, China), 10 mg/kg/d; CsA (Novartis, Germany), 50 mg/kg/2d; and CYP (Baxter, Germany), 25 mg/kg/4d. MMF (Roche, Germany) was diluted in saline, and a gavage was administered 1 week prior to HI at a dose of 20 mg/kg/d [12]–[16].

### HI

Mice received an HI of pAAV/HBV1.2 (kindly provided by Prof. Chen PJ, Graduate Institute of Clinical Medicine, College of Medicine, National Taiwan University) as described previously [8].

### Adoptive immune transfer

The immunized donor mice were injected intramuscularly with 100 µg of pHBc as described previously [9]. Splenocytes from the immunized or naïve donor mice were prepared as described previously [8]. HBV-persistent mice treated with CsA for 10 weeks were left as control or injected intravenously with 1×10^8^ splenocytes from the immunized or naïve donor mice at week (w) 12 after HI.

### Detection of HBsAg, HBsAb, HBcAb, and HBV DNA in the mouse sera

The serum samples were diluted 1∶10 with Diluent Universal (Roche Diagnostics, Switzerland). Hepatitis B virus surface antigen (HBsAg), hepatitis B virus surface antibody (HBsAb), and hepatitis B virus core antibody (HBcAb) were detected using the electrochemiluminescence immunoassay (ECLIA) system (Roche Diagnostics, Switzerland), according to the manufacturer's instructions. The percentage of inhibition of HBcAb was calculated with the formula 100× (index calibrator mean rate - sample rate)/index calibrator mean rate. The HBV DNA levels were quantified by a real-time polymerase chain reaction (PCR) using SYBR Green dye as described previously [9]. The following primers were used: forward primer, 5′-CTG CAT CCT GCT GCT ATG-3′ (nt 408–425), and reverse primer, 5′-CAC TGA ACA AAT GGC AC-3′ (nt 685–701), based on the reference sequence with GenBank accession number AY220698. A serum sample containing a known concentration of HBV DNA was used as a positive control.

### Southern blot analysis of HBV replication intermediates in the liver tissues

The total DNA was extracted from the liver samples using the Tissue DNA Kit (Omega, USA) and analyzed by Southern blotting using a ^32^P-labeled, full-length HBV probe, as described previously [17].

### Immunohistochemistry (IHC)

The liver samples were collected and embedded in paraffin routinely. The sections were stained with rabbit anti-HBV core antigen (HBcAg) polyclonal antibody (Dako, Japan) and visualized by the DAKO EnVision™ Detection Systems (Dako, Japan), according to the manufacturer's instructions.

### Enzyme-linked immunospot assay (ELISPOT)

The splenocytes were separated by Ficoll density-gradient centrifugation according to the manufacturer's instructions (Dakewe, China). The splenocytes were then cultured at a density of 2×10^5^ cells per well in the presence of recombinant HBsAg (rHBsAg), recombinant HBcAg (rHBcAg), or an unrelated CMV peptide (all from ProSpec-Tany, China) at a concentration of 0.5 µg/ml. Splenocytes cultured in medium or in 5 µg/ml of ConA (Invitrogen, USA) served as the negative or positive controls, respectively. The ELISPOT assay was performed using the Mouse IFN-γ precoated ELISPOT Kit (Dakewe, China) according to the manufacturer's instructions. The spots were counted using the ImmunoSpot analyzer and the supporting ImmunoSpot software (Cellular Technology, USA). The results are presented as spot-forming cells per 2×10^5^ cells.

### The expression levels of CD molecules and cytokines in the liver

The mRNA levels of the CD molecules and cytokines were measured by real-time reverse-transcription PCR using SYBR Green dye. Briefly, total RNA was extracted from the mouse liver samples using Trizol reagent (Invitrogen, USA). The relative mRNA levels for each parameter were analyzed using the QuantiFast SYBR Green PCR kit and QuantiTect Primers (both from Qiagen, Germany) according to the manufacturer's instructions. The quantification was performed using the Pfaffl method [18] by calculating the copy numbers from the ct-value for each gene per sample. The β-actin mRNA levels were calculated in the same manner and used for normalization. The expression levels of the selected genes were given as the copy number/100,000 copies of β-actin mRNA.

### Statistical analysis

The statistical analysis was conducted using SPSS software version 12.0 (SPSS Inc, USA). Differences between two groups or among multiple groups were analyzed by the two-tailed unpaired Student's t test and one-way ANOVA with Tukey's multiple comparison tests, respectively. A P value <0.05 was considered statistically significant.

## Results

### Four immunosuppressants have different effects on HBV replication

To ensure the comparable transfection efficiency among immunosuppressant-treated and control mice, the levels of HBV DNA and HBsAg in the sera were measured at day (d) 4 post HI. The levels of HBV DNA and HBsAg did not significantly differ between the saline-treated or immunosuppressant-treated mice at d4 (Fig. S1A and S1B).

**Figure 1:**
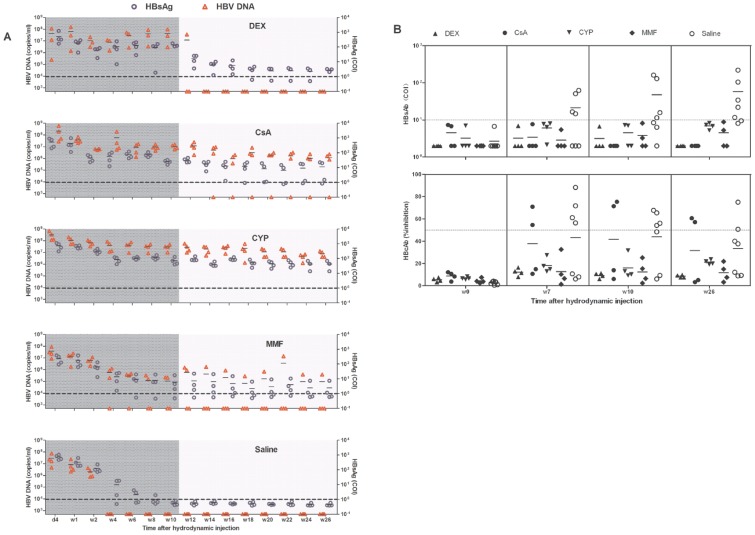
HBV persistence in mice receiving HI and immunosuppressive treatment. Mice were treated with saline or the immunosuppressive drugs DEX, CsA, CYP, or MMF from 1 week before the HI to 10 weeks after the HI, and they received the HI at w0. (A) The levels of serum HBsAg (circle) and HBV DNA (triangles) in the mice after the HI were detected using ECLIA, with the cut off values of 1.0 COI (dashed line) and 5×10^2^ copies/ml, respectively. (B) The production of HBsAb and HBcAb in the mice was measured using ECLIA assay at w0, w7, w10, and w26 after HI. The cut off value of the HBsAb assay was 10 IU/L. The positivity of the HBcAb test was defined as inhibition >50%.

To analyze the influence of different immunosuppressants on HBV replication, HBsAg and HBV DNA levels were dynamically measured during the treatment duration. HBsAg and HBV DNA in sera from mice treated with DEX, CsA, or CYP maintained at the high levels of approximately 100 cut off index (COI) and 10^6–7^ copies/ml, respectively. The sera levels of HBV DNA and HBsAg in the MMF-treated mice decreased gradually and became negative within 4 weeks post HI in 1 animal, and the HBV DNA was negative in another animal at w8 post HI (Fig. 1A). By contrast, the HBV replication in the BALB/c mice usually ceased within a few weeks, the HBV DNA became negative within 4 weeks, and the HBsAg was eliminated within 6 weeks in mice treated with saline (Fig. 1A).

To measure if transient immunosuppressant treatment can induce long-term HBV persistence, the HBsAg and HBV DNA levels were followed for another 16 weeks after the immunosuppressants were discontinued at w10. DEX-treated mice became negative for HBV DNA within 4 weeks after DEX withdrawal, while the HBsAg levels decreased rapidly and were maintained at less than 10 COI until 26 weeks post HI (Fig. 1A). In the majority of the CsA-treated mice, the HBsAg and HBV DNA levels were maintained until w26 at approximately 10 COI and 10^6^ copies/ml, respectively (Fig. 1A). The CYP-treated mice were persistently positive for HBsAg and HBV DNA until 26 weeks post HI at high levels of approximately 100 COI and 10^6–7^ copies/ml, respectively (Fig. 1A). The HBV DNA became negative within 4 weeks after MMF discontinuation in 1 animal; in addition, the HBsAg decreased slowly and became negative in the majority of MMF-treated mice, whereas the HBsAg and HBV DNA in 1 animal remained positive at w26 (Fig. 1A).

To explore the influence of immunosuppression on the HBV antibody response, HBcAb and HBsAb were measured at d0, w7, w10 (the end of treatment), and w26 (the end of follow-up). At d0 (before the HI), HBcAb and HBsAb were negative in all the animals. The HBcAbs were detectable at w7, w10, and w26 both in the control mice and in the CsA-treated mice but were negative in the DEX-, CYP-, and MMF-treated mice. The HBsAbs were detectable at w7, w10, and w26 in the control mice but were negative in all the immunosuppressant-treated mice at w7, w10, and w26 (Fig. 1B); some mice even became negative for the HBsAg and HBV DNA at w26 (Fig. 1A).

### The treatment duration of CsA has an impact on HBV persistence

**Figure 2:**
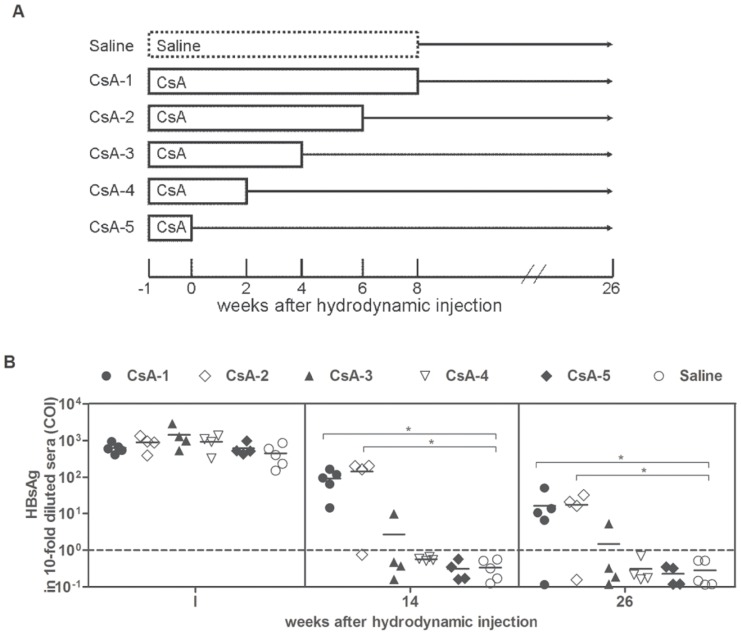
HBV persistence in mice receiving short-term CsA treatment. (A) Schema of the CsA treatment. The mice were treated with saline or CsA from w1 before the HI to w0, w2, w4, w6, and w8 after the HI. (B) The serum HBsAg levels in the mice at the indicated time points after the HI were detected. Cut off value (dashed line). * P<0.05.

To investigate the time of transient immunosuppressant treatment necessary for inducing HBV persistence, CsA was selected to treat the HI mice from 1 week before the HI to different time points post HI (Fig. 2A). Mice treated with saline served as controls. At w1 post HI, the HBsAg levels were comparable in the control and CsA-treated mice, indicating that the mice in the two groups had a comparable transfection efficiency (Fig. 2B). At w14, the control mice and the mice treated with CsA for less than 4 weeks post HI were HBsAg negative, while the majority of mice treated with CsA until w6 and w8 post HI were HBsAg positive (Fig. 2B). At the end of the observation period (at least 18 weeks after CsA withdrawal), the HBsAg remained positive in the majority of mice treated with CsA until w6 and w8 (Fig. 2B). These data indicated that CsA treatment from 1 week before HI to more than 6 weeks after HI causes HBV persistence for at least 6 months.

**Figure 3:**
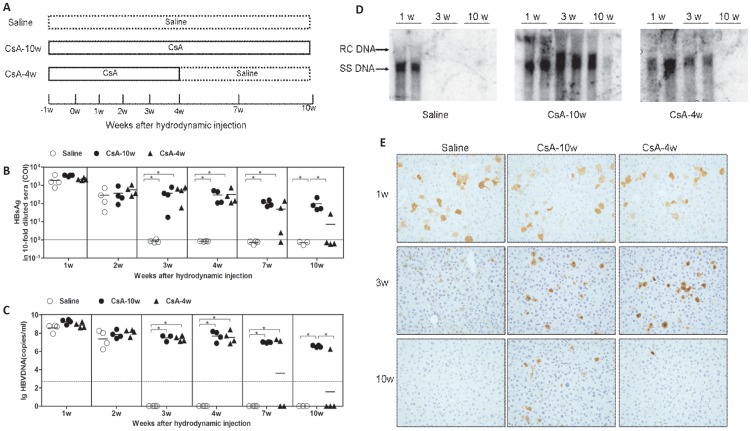
HBV replication and gene expression in CsA- or saline-treated mice after HI. (A) Schema of the treatment. The mice were treated with saline (Saline) or CsA from 1 week before the HI to 4 weeks (CsA-4w) or 10 weeks (CsA-10w) after the HI. The levels of serum HBsAg (B) and HBV DNA (C) in the mice at the indicated time points were detected. The cut off values of HBsAg and HBV DNA are displayed as solid and dashed lines, respectively. Liver samples were collected from the mice (n≥3) at the indicated time points. The HBV replicative intermediates (D) and HBcAg (E) in the liver were analyzed by Southern blotting and IHC (magnification 200×), respectively.

We further analyzed the HBV replication and gene expression in the mice receiving saline (Saline) or CsA from 1 week before HI to 4 or 10 weeks post HI (referred as CsA-4w and CsA-10w, respectively) (Fig. 3A). Consistently, the saline-treated mice rapidly cleared HBsAg and HBV DNA from the peripheral, while HBsAg and HBV DNA persisted during the entire observation period in the CsA-10w mice. The HBsAg and HBV DNA levels in the CsA-4w mice decreased after the CsA withdrawal and were undetectable in three of four mice at w10 (Fig. 3B and 3C). The HBV replicative intermediates and HBcAg in the mouse liver were examined by Southern blot analysis and IHC (Fig. 3D and 3E). Consistent with HBV markers in the peripheral blood, HBV replicative intermediates and HBcAg in the liver were present at w1 in the livers from all the mice in the three groups and became undetectable at w3 and w10 in the saline-treated mice and the CsA-4w mice, respectively, while remaining detectable at w10 in the CsA-10w mice.

### CsA treatment suppresses the T cell response in the liver

**Figure 4:**
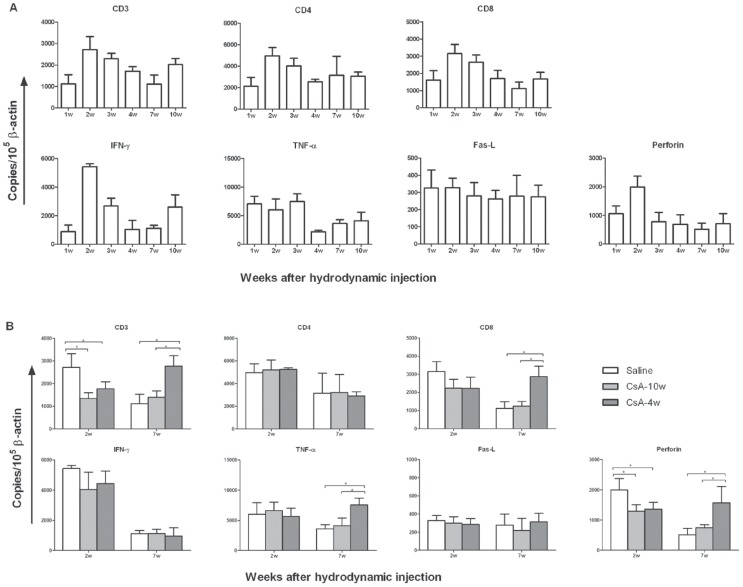
The influence of CsA treatment on the intrahepatic T cell response in mice after the HI. The mice were treated with saline or CsA from 1 week before the HI to 4 weeks (CsA-4w) or 10 weeks (CsA-10w) after the HI. The liver samples were collected from the mice (n≥3) at the indicated time points. The T cell markers, antiviral cytokines, and cytotoxic molecules were analyzed by real-time RT-PCR and are shown as the mean expression levels relative to 10^5^ β-actin mRNA copies. (A) Dynamic changes in the intrahepatic expression of the T cell-related molecules in the saline-treated mice. (B) The expression of the T cell-related molecules was compared in livers from the saline- or CsA-treated mice at w2 and w7. * P<0.05.

During HI, the HBV plasmid is predominantly transfected into the liver and leads to HBV replication in the mouse liver, which mimics HBV replication in the human liver. Thus, the intrahepatic mRNA levels of the T cell markers (e.g., CD3 and CD8), the antiviral cytokines (e.g., IFN-γ and tumor necrosis factor alpha [TNF-α]), and the cytotoxic molecules (e.g., Fas ligand [Fas-L] and perforin) were quantified by real-time reverse-transcription PCR (Fig. 4). We first analyzed the dynamic changes of these molecules in the livers from saline-treated mice. The results revealed that the levels of CD3, CD8, IFN-γ, Fas-L, and perforin were significantly elevated at w2 (accompanied by the decreased HBsAg and HBV DNA levels in the serum) and that the virus was ultimately eliminated at w3 from both the serum and the liver (Fig. 4A). Subsequently, we compared the expression of these molecules in the livers from the mice receiving saline or CsA at w2 and w7, which were the time points with decreased HBV replication in the saline-treated mice or the CsA-4w mice. At w2, all the molecules elevated in the saline-treated mice were suppressed in the CsA-treated mice, while only the decline of CD3 and perforin were statistically significant (Fig. 4B). At w7, the discontinuation of the CsA treatment at w4 led to a significant increase in the levels of CD3, CD8, TNF-α, and perforin, coincident with the declined HBV replication at w7 and HBV clearance at w10 (Fig. 4B).

### Adoptive immune transfer controls HBV replication in HBV-persistent mice

**Figure 5:**
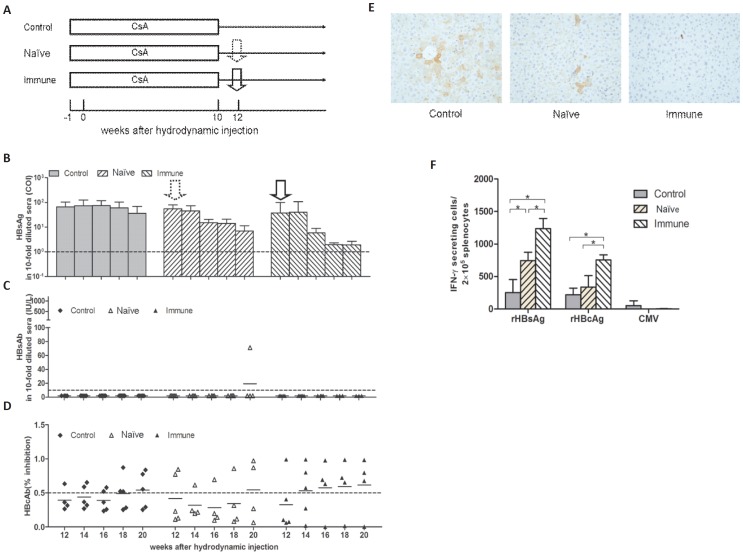
Adoptive immune transfer in mice with HBV persistence induced by CsA treatment. (A) Schema of the treatment. The mice were treated with CsA from 1 week before the HI to 10 weeks after the HI. Two weeks later, the mice were adoptive transferred with the splenocytes from naïve or pHBc-immunized mice. The levels of serum HBsAg (B), HBsAb (C), and HBcAb (D) in the mice were detected at the indicated time points. The cut off values of the HBsAg, HBsAb, and HBcAb are shown as dashed lines. (E) Liver samples were collected from the mice (n≥3) at week 24 post HI (12 weeks after the adoptive transfer), and intrahepatic HBcAg was detected by IHC (magnification 200×). (F) Splenocytes were prepared from the mice (n≥3) at week 24 post HI (12 weeks after the adoptive transfer), and pulsed with rHBsAg and rHBcAg, IFN-γ-producing cells were analyzed by ELISPOT, as described above. * P<0.05.

Transient CsA treatment leads to the suppression of the intrahepatic T cell response and allows long-term HBV persistence in this mouse model. Adoptive transfer of immune cells from individuals with immunity to the HBV infection boosts the host immune responses to HBV, as published in a few clinical and animal studies [19], [20]. Therefore, we asked whether the long-term HBV persistence induced by transient CsA treatment could be terminated by the adoptive immune transfer. Three groups of mice received CsA treatment for 10 weeks post HI. At w12, the mice received splenocytes from naïve mice (Naïve) or mice immunized with the HBcAg expression plasmid pHBc (Immune) or were left as controls (Control) (Fig. 5A). The transfer of splenocytes from the immune mice led to a significant decrease in the levels of serum HBsAg and liver HBcAg and enhanced the HBcAb responses and the cellular responses to HBcAg in the recipient mice (Fig. 5B, 5D, 5E, and 5F), whereas HBsAb was remained undetectable in the recipient mice (Fig. 5C). Serum HBsAg and liver HBcAg were persistently detected in the control and naïve mice (Fig. 5B and 5E). All the mice in the control and naïve groups (except for one naïve mouse) remained negative for HBsAb (Fig. 5C). HBcAb and the cellular responses to HBcAg were unchanged in the mice from both groups (Fig. 5D and 5F).

## Discussion

Using a mouse model with acute hepatitis B replication, we demonstrated that treatment with four immunosuppressants had different effects on HBV replication. HBV replication was not eliminated in the immunosuppressant-treated mice and was maintained at a high level in mice treated with DEX, CsA, and CYP, but not with MMF, although the mice receiving the same regimen of DEX, CsA, CYP, and MMF treatment exhibited a comparable, extended survival time of the skin grafts (Fig. S2). After the withdrawal of immunosuppressant treatment, HBV was persistent at a high level in the CsA- and CYP-treated mice, and at a low level in the DEX-treated mice. Furthermore, the early (at w4 post HI) withdrawal of the CsA treatment restored the suppressed intrahepatic CD8 T cell response and led to subsequent HBV clearance. Finally, adoptive transfer with the splenocytes from donor mice immunized with HBcAg expression plasmid led to HBV suppression in these HBV-persistent mice.

In the present study using the HBV HI mice model, the four immunosuppressants had different effects on HBV replication in vivo, consistent with the effect of immunosuppressants on HBV replication in vitro, e.g. CsA suppressed HBV replication while MMF could enhance or suppress HBV replication depending on the content of HBV infection[21], [22]. In addition, this is comparable with the situation of HBV reactivation during organ transplantation or chemotherapy, although any form of immunosuppressive treatment can result in HBV reactivation, certain drugs are associated with a higher risk than others are, e.g., steroids are most frequently associated with HBV reactivation [23]. This may be associated with the different effects on the virus-specific T cell response, e.g. tacrolimus, a calcineurin inhibitor, was potent in inhibiting CMV-specific Th1 cytokines, whereas MMF preferentially inhibited CMV-specific Th2 cytokines [24]. Furthermore, MMF was recently found to inhibit the activity of inosine monophosphate dehydrogenase (resulting the decrease of intracellular levels of guanosine nucleotide pools) and to augment interferon-stimulated gene expression[25]. These properties of MMF may also contribute to the reduction of HBV DNA in MMF-treated mice.

Although immunosuppressants are beneficial for increasing the long-term survival in patients with organ transplants, the lifelong administration of immunosuppressants also yields numerous side effects, e.g., HBV reactivation and recurrence (discussed in this article) and the high risk of the infection with other pathogens. Recently, several studies have attempted to evaluate the impact of the minimization and complete withdrawal of immunosuppressants in liver transplantation [26]. The immunosuppressants were successfully discontinued in approximately 20% of carefully selected recipients (e.g., those with a longer duration after the transplantation), indicating that long-term immunosuppression led to tolerance against the transplanted liver. Interestingly, in the present study, the discontinuation of immunosuppressive treatment did not always result in the restoration of immunological functions and subsequent HBV clearance, which may also suggest that immunosuppressive treatment using the selected drugs for a sufficient length of time causes tolerance against HBV.

CD8+ T cells, particularly intrahepatic HBV-specific CD8+ T cells, play a critical role in HBV clearance [27]. The frequencies of HBV-specific CD8 T cells were generally extremely low in Balb/C mice that received an HI of pAAV/HBV1.2 and could not be properly measured by intracellular staining and flow cytometry analysis (Fig. S3). Therefore, we analyzed the intrahepatic mRNA expression of the molecules closely related to the CD8 T cell immune response, including the T cell markers (e.g., CD3 and CD8), the antiviral cytokines (e.g., IFN-γ and TNF-α), and the cytotoxic molecules (e.g., Fas-L and perforin). The expression of CD3, CD8, IFN-γ, and perforin was increased after the HBV transfection, accompanied by a decrease in the HBV replication and the subsequent HBV clearance, which was consistent with the findings in the woodchuck liver with acute woodchuck hepatitis virus infection [28]. Although IFN-γ and perforin can also be produced by NK cells, NK-cell depletion by an anti-asialo antibody could not prolong the HBV replication in these immunocompetent mice (data not shown), indicating that the HBV clearance in these immunocompetent mice was mainly mediated by IFN-γ- and perforin-dependent CD8 T cell responses. This result is not completely consistent with a previous study using knockout mice deficient in the numerous cellular or molecular effectors. Using the knockout mice, those researchers found that CD8 T cells mediated HBV clearance in a Fas-dependent but perforin-independent manner [29]. CsA treatment extended the HBV replication and suppressed the intrahepatic expression of CD3, CD8, IFN-γ, and perforin; moreover, early CsA withdrawal restored the intrahepatic expression of CD3, CD8, TNF-α, and perforin and led to HBV clearance from the liver, thereby providing more evidence regarding CD8 T cell response-mediated HBV clearance in this immunocompetent mice model. It should be noted that the alteration of the mRNA expression may be a result of different events including the general systemic reduction of T cell responses, reduced liver-infiltration of specific T cells, the different cell proliferation status in the liver, and the alteration of the expression profiles at the single cell level.

The specific cellular immune responses to the hepadnaviral core antigen were considered critical for controlling the HBV infection [30], [31] and WHV infection in woodchucks [32]. Here, the adoptive transfer of splenocytes from mice immunized with an HBcAg expression plasmid effectively enhanced the cellular responses to the HBV proteins and suppressed HBV replication in the HBV-persistent mice. This result is consistent with the clinical findings in patients with liver transplants and bone marrow transplants [33]. Adoptive immune transfer might be a promising immunotherapeutic approach in patients with HBV recurrence after liver transplantation.

Taken together, we demonstrated that immunosuppressive treatment can extend HBV replication and cause long-term HBV persistence even after immunosuppressant withdrawal, and HBV replication under immunosuppression depends on the immunosuppressive drugs and the treatment duration. These findings may be helpful for individualized treatment with immunosuppressive drugs in patients at high risk for HBV reactivation and recurrence. For this purpose, other immunosuppressive agents, such as tacrolimus and rapamycin, must also be evaluated in this system. We also demonstrated that an impaired intrahepatic CD8 T cell response may be responsible for HBV persistence under immunosuppression and that the restoration of the T cell response leads to HBV clearance or suppression in this HBV-persistent mouse model, thereby mimicking the HBV infection in humans. Therefore, an HBV mouse model based on HI is suitable for studying the HBV infection under immune suppression and for evaluating and optimizing the approaches for the prophylaxis and treatment of HBV reactivation and recurrence under immunosuppressive therapy.

## Supporting Information

Figure S1 **The comparable transfection efficiency in mice receiving saline or immunosuppressants after HI.** Mice were treated with saline or the immunosuppressive drugs DEX, CsA, CYP, or MMF from 1 week before the HI, and the levels of serum HBsAg (A) and HBV DNA (B) were compared at d4 post HI. P>0.05.
(TIF)

Figure S2 **Skin-graft survival times in the mice treated with immunosuppressive drugs.** BALB/c mice were treated with saline or the immunosuppressive drugs DEX, CsA, CYP, or MMF from 1 week before skin transplantation to the end of follow-up. The skin grafts of the donor mice (strain C57BL/6, male, 8–10 weeks of age) were transplanted to the lateral thorax of the recipient BALB/c mice at w0. The grafts were inspected daily until rejection, which was defined as >90% necrosis of the graft epithelium. n≥5, *P<0.05.
(TIF)

Figure S3 **The frequencies of HBcAg-specific CD8 T cells in mice received HI of pAAV/HBV1.2.** Three mice were sacrificed at w2 after the HI of pAAV/HBV1.2, and splenocytes were isolated and incubated with the peptide HBc87–95 (ProSpec-Tany, China) for 5 hours at the concentrations of 2µg/ml. Spenocytes from a mouse were stimulated with phorbol myristol acetate (PMA)/ionomycin (both from Invitrogen, USA) at the concentration of 400 ng/ml (PMA) and 10µg/ml (ionomycin), and served as positive control. The cells were harvested and stained with PE-labeled CD8 antibody and APC-labeled IFN-γ antibody (both from BD Pharmingen, USA) and analyzed by flow cytometry. d
(TIF)
