# Supplementary figures and images for: Immunosuppressive Drugs Modulate the Replication of Hepatitis B Virus (HBV) in a Hydrodynamic Injection Mouse Model
Source: PLoS One. 2014 Jan 21;9(1):e85832. doi: 10.1371/journal.pone.0085832 (PMC3897536; doi:10.1371/journal.pone.0085832)

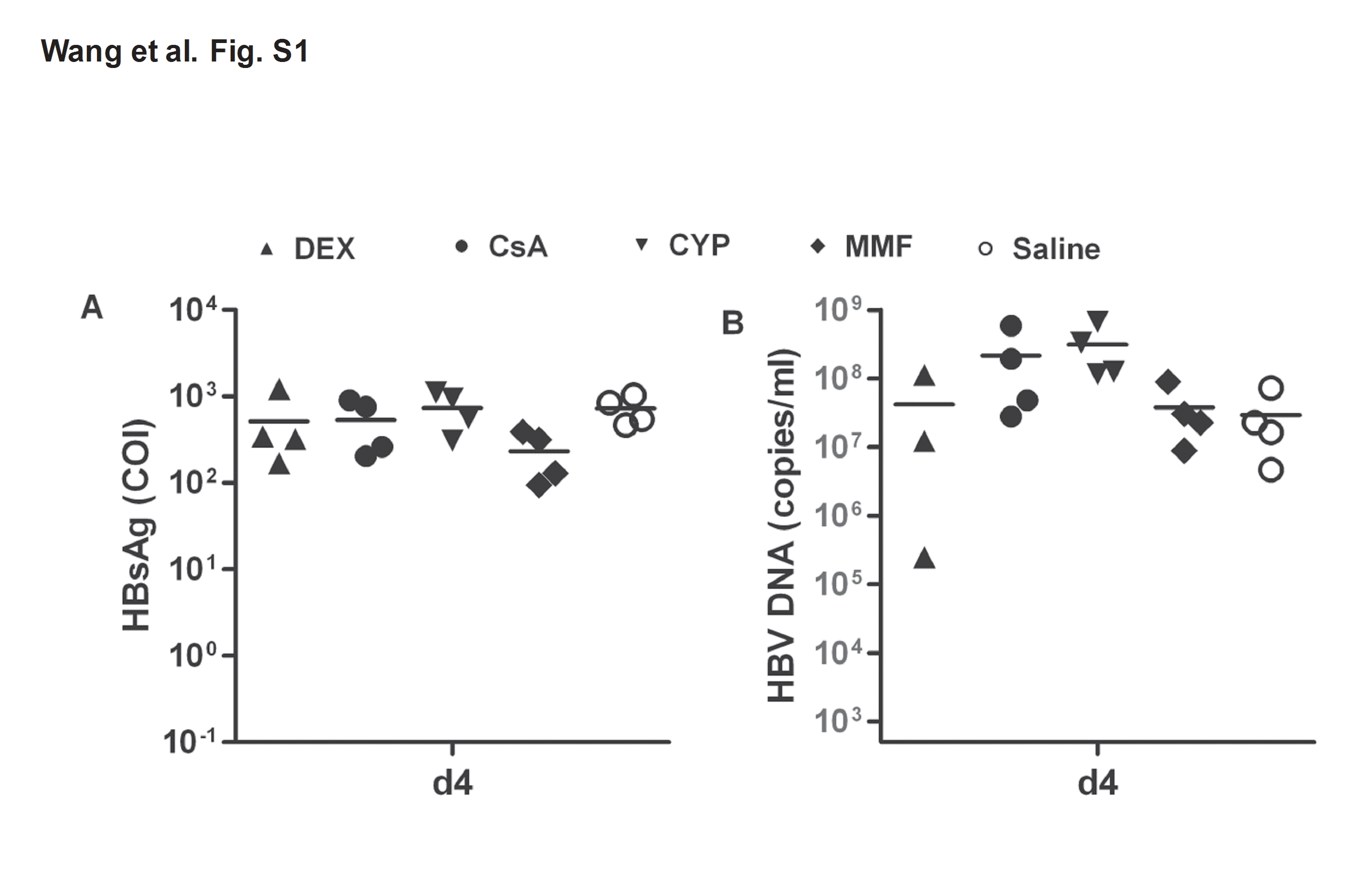

Supplement: Figure S1 — The comparable transfection efficiency in mice receiving saline or immunosuppressants after HI. Mice were treated with saline or the immunosuppressive drugs DEX, CsA, CYP, or MMF from 1 week before the HI, and the levels of serum HBsAg (A) and HBV DNA (B) were compared at d4 post HI. P>0.05. (TIF) [file pone.0085832.s001.tif]

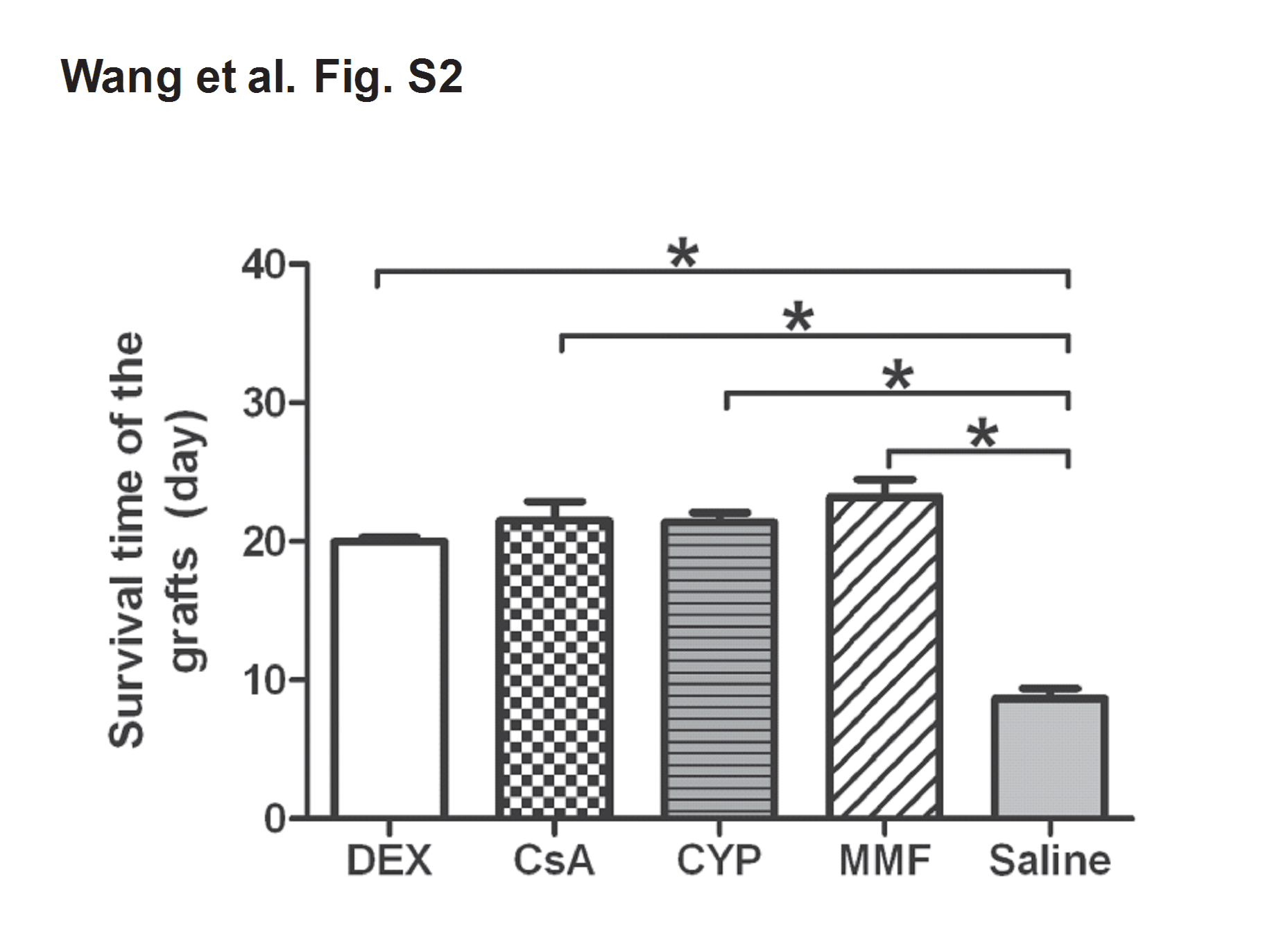

Supplement: Figure S2 — Skin-graft survival times in the mice treated with immunosuppressive drugs. BALB/c mice were treated with saline or the immunosuppressive drugs DEX, CsA, CYP, or MMF from 1 week before skin transplantation to the end of follow-up. The skin grafts of the donor mice (strain C57BL/6, male, 8–10 weeks of age) were transplanted to the lateral thorax of the recipient BALB/c mice at w0. The grafts were inspected daily until rejection, which was defined as >90% necrosis of the graft epithelium. n≥5, *P<0.05. (TIF) [file pone.0085832.s002.tif]

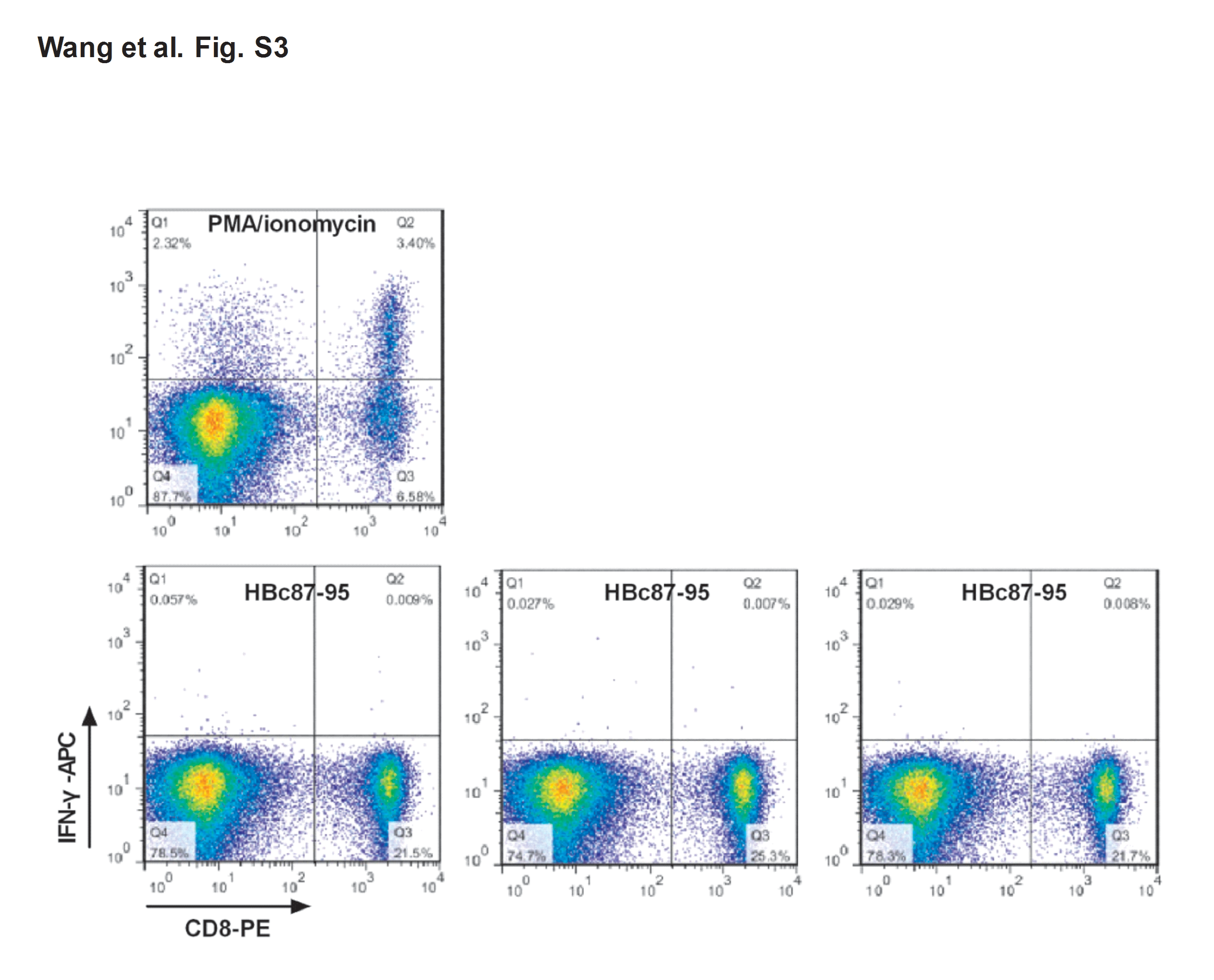

Supplement: Figure S3 — The frequencies of HBcAg-specific CD8 T cells in mice received HI of pAAV/HBV1.2. Three mice were sacrificed at w2 after the HI of pAAV/HBV1.2, and splenocytes were isolated and incubated with the peptide HBc87–95 (ProSpec-Tany, China) for 5 hours at the concentrations of 2µg/ml. Spenocytes from a mouse were stimulated with phorbol myristol acetate (PMA)/ionomycin (both from Invitrogen, USA) at the concentration of 400 ng/ml (PMA) and 10µg/ml (ionomycin), and served as positive control. The cells were harvested and stained with PE-labeled CD8 antibody and APC-labeled IFN-γ antibody (both from BD Pharmingen, USA) and analyzed by flow cytometry. d (TIF) [file pone.0085832.s003.tif]
